# Using Different Cutoffs to Define Tinnitus and Assess Its Prevalence—A Survey in the Dutch General Population

**DOI:** 10.3389/fneur.2021.690192

**Published:** 2021-08-11

**Authors:** Maaike M. Rademaker, Adriana L. Smit, Anne E. M. Brabers, Judith D. de Jong, Robert J. Stokroos, Inge Stegeman

**Affiliations:** ^1^Department of Otorhinolaryngology, Head and Neck Surgery, University Medical Center Utrecht, Utrecht, Netherlands; ^2^University Medical Center Utrecht Brain Center, University Medical Center Utrecht, Utrecht, Netherlands; ^3^Nivel, Netherlands Institute for Health Services Research, Utrecht, Netherlands; ^4^Care and Public Health Research Institute, Maastricht University, Maastricht, Netherlands; ^5^Department of Ophthalmology, University Medical Center Utrecht, Utrecht, Netherlands; ^6^Epidemiology and Data Science, Amsterdam University Medical Centers, University of Amsterdam, Amsterdam, Netherlands

**Keywords:** tinnitus, prevalence, definition, tinnitus heterogeneity, general population

## Abstract

**Introduction:** Tinnitus prevalence numbers in the literature range between 5 and 43%, depending on the studied population and definition. It is unclear when tinnitus becomes pathologic.

**Objectives:** To assess the tinnitus prevalence in the Dutch general population with different cutoffs for definition.

**Methods:** In this cross-sectional study, a questionnaire was sent to a sample (*n* = 2,251) of the Nivel (Netherlands Institute for Health Services Research) Dutch Health Care Consumer Panel. Three questions were asked to assess the presence of tinnitus, duration, and frequency of the complaint. We classified people as having pathologic tinnitus when participants experienced it for 5–60 min (daily or almost daily or weekly), or tinnitus for >60 min or continuously (daily or almost daily or weekly or monthly), so tinnitus impact on daily life was measured with the Tinnitus Functional Index (TFI) and a single-item question. Answers were stratified to mid-decade years of age. Prevalence numbers were weighted by gender and age to match the Dutch population.

**Results:** Nine hundred thirty-two of 2,251 participants (41%) filled out the questionnaire. The median age was 67.0 (*IQR* 17) years. Three hundred thirty-eight of 932 (36%) experienced tinnitus for an undefined amount of time during the last year. Two hundred sixteen of 932 (23%) met our definition of having pathologic tinnitus (21% when weighted for age and gender). The median TFI score for all pathologic tinnitus participants was 16.6 (*IQR* 21.8). A percentage of 50.4% of the pathologic tinnitus participants had a TFI in the range 0–17, which can be interpreted as not a problem.

**Conclusion:** Twenty-three percent (unweighted) or 21% (weighted) of our sample met our definition of pathologic tinnitus, which was based on a combination of duration and frequency over the last year. The TFI score of 47.7% of the pathologic tinnitus participants is ≥18. This indicates that they consider the tinnitus to be at least “a small problem” [11.1% (unweighted) or 8.9% (weighted) of the total study group]. This study illustrates the difficulties with defining pathologic tinnitus. In addition, it demonstrates that tinnitus prevalence numbers vary with different definitions and, consequently, stresses the importance of using a uniform definition of tinnitus.

## Introduction

Till date, the prevalence of tinnitus in the general population remains uncertain. In a systematic review conducted in 2016, a wide range in tinnitus prevalence numbers was found in included studies, with numbers varying between 5.1 and 42.7% in the adult population (1–3). The variation in numbers is mainly believed to be caused by the use of different definitions of tinnitus. The phenomenon of tinnitus is clearly described in literature as the experience of a sound, in the absence of an external stimulus (4). Still, the authors of the systematic review identified eight variations on screening questions to identify those having tinnitus. This varied from tinnitus lasting for more than 5 min at a time or the experience of tinnitus within the last year. However, besides criteria of time elements, there are multiple components that could contribute to a definition. The authors argue that, for example, the impact of tinnitus on daily life could be part of the definition (1), since the mere presence of tinnitus does not necessarily mean the individual person experiences it as pathologic or distressing (4). At this moment there is no consensus on when tinnitus becomes so distressing that it becomes pathologic, or the individual starts, for example, to seek help.

Knowledge about the prevalence of a disease is important for the organization of healthcare and prevention of the condition (5). Moreover, in the conceptual analysis “Why is there no cure for tinnitus” published in 2019, several other consequences were related to the lack of more detailed knowledge about prevalence numbers such as the lack of improvement in pharmacological therapies (6). Due to the absence of prevalence information, companies are not informed about the potential market for their future product and therefore do not develop a product for patients (6). These issues urge the need to assess the prevalence of tinnitus in the general population by usage of a clear description of the experienced symptoms.

In order to elucidate the tinnitus prevalence, we have designed this study. We wanted to assess tinnitus prevalence in a general population sample. Next, to tackle the issues of defining tinnitus, we asked several questions, with different cutoffs, rather than one general screening question. These included questions on tinnitus presence, but also on the impact of tinnitus on daily life. Our primary aim was, therefore, to assess the prevalence of tinnitus in the Dutch general population with different cutoffs for its definition.

## Methods

This paper was written according to the Strengthening the Reporting of Observational Studies in Epidemiology (STROBE) statement (7).

### Study Design and Population

This is a cross-sectional study of a cohort of people aged 18 years and older of the Dutch population. Data was prospectively collected with a questionnaire sent to a sample of panel members of the Dutch Health Care Consumer panel (DHCCP) of Nivel (the Netherlands institute for health services research) (8).

The goal of the Dutch Health Care Consumer Panel is to measure, at national level, opinions on and knowledge about healthcare and the expectations and experiences with healthcare. The Consumer Panel is a so-called access panel. An access panel consists of a large number of persons who have agreed to answer questions on a regular base. In addition, many background characteristics of these persons (for example age, level of education, income, self-reported general health) are known. At the time of this study (January 2020), the panel consisted of approximately 12,000 people aged 18 years and older. From the access panel, samples can be drawn for every separate survey. It is not possible for people to sign up on their own initiative. The panel is renewed on a regular basis. Renewal is necessary to make sure that members do not develop specific knowledge of, and attention for, healthcare issues, and that no “questionnaire fatigue” occurs. Moreover, renewal compensates for panel members who, for example, have died or moved without informing us about the new address (8).

This study is a smaller part of a larger study on tinnitus characteristics, risk factors, and healthcare usage. The questionnaire sample therefore consisted of all panel members (*N* = 2.291) of the Consumer Panel who gave permission to combine their answers of the survey with healthcare consumption data as registered by their general practitioner (9). The participants of the DHCCP received a questionnaire by postal mail, and online, depending on the preference of the panel member. The postal questionnaire was sent on January 14, 2020, with one postal reminder sent on January 30. The online questionnaire was sent on January 16, 2020; two electronic reminders were sent on January 23 and January 30, 2020. The questionnaire closed on February 14, 2020. No further actions were undertaken to optimize the response rate for this study specifically. In general, all panel members are kept involved by newsletters. The study reports on a part of the data collected in the questionnaire.

### Outcome Assessment

#### Questionnaire

##### Tinnitus Presence

The presence of tinnitus was assessed with three questions, based on the studies by McCormack et al., Baguley et al., and Langguth et al. and expert opinion (1, 10, 11). First, all participants were asked the question whether they had experienced tinnitus in the last year. Tinnitus was described as the following: *Tinnitus is the hearing of e.g., a beep, whistle, sissing, zoom, or another sound without the actual presence of the sound in your surroundings. This can last for a very short amount of time or a whole day*. If participants responded positively (yes) on that question, they were asked two follow-up questions. The first inquired about the time-related characteristics of the tinnitus (tinnitus lasting <5 min, 5–60 min, >60 min, or continuously) and the second about frequency of the experienced sound (daily or almost daily, weekly, monthly, less than once a year). To interpret the outcome, we classified people as having pathologic tinnitus when they were experiencing tinnitus for 5–60 min (daily or almost daily or weekly) and >60 min or continuously (daily or almost daily or weekly or monthly).

##### Impact of Tinnitus

Participants that met the definition of pathologic tinnitus were asked about the impact of tinnitus on daily life measured with the Tinnitus Functional Index (TFI) (12, 13). The TFI consists of 25 questions, each with an 11-point Likert scale. The TFI creates a score from 0 (not a problem) to 100 (a very big problem), which can be subdivided into five categories; namely, scores ranging between 0 and 17 can be interpreted as not a problem, 18–31 as a small problem, 32–53 as a moderate problem 54–72 as a big problem, and 73–100 as a very big problem (14). Furthermore, the TFI consists of eight subscales to measure the impact of tinnitus on intrusiveness, sense of control, cognition, sleep, hearing, relaxation, quality of life, and emotions. The questionnaire was first developed in English and validated before translation to Dutch in 2014. The Dutch translation has a high internal consistency with a Cronbach's alpha of 0.91 (13).

##### Subjective Problem

The question: “*how big a problem is your tinnitus at this moment?”* was asked to those with pathologic tinnitus. Answer options were “*no problem*,” “*small problem*,” “*reasonable problem*,” “*large problem*,” or “*very large problem*.”

### Data Handling and Ethics

Data are analyzed anonymously, and the privacy of the panel members is guaranteed, as is described in the privacy policy of the Dutch Health Care Consumer Panel. This complies with the General Data Protection Regulation (GDPR). According to Dutch legislation, neither obtaining informed consent nor approval by a medical ethics committee is obligatory for conducting research through the panel (CCMO, 2020) (8). The Medical Research Ethics Committee (MREC) of the University Medical Center Utrecht (UMC Utrecht) confirmed on November 20, 2019, that the Medical Research Involving Human Subjects Act (WMO) does not apply to this study and that therefore official approval by the MREC is not required under the Human Subjects Act (MREC local protocol number 19-745).

### Statistical Analysis

Statistical analyses were performed with SPSS version 25.0.0.2 Normality was visually assessed. Frequencies, medians, and interquartile range (*IQR*) were calculated. Prevalence data and the subjective problem of tinnitus were stratified per mid-decade groups. Total TFI scores were calculated for those with pathologic tinnitus and stratified per mid-decade groups. Tinnitus Functional Index categories and subscales were calculated. The sample was not representative in terms of age for the Dutch population. To give a more precise estimate of the prevalence numbers, we corrected the prevalence numbers of pathologic tinnitus with a weight factor by age and gender. The weight factors ranged from 0.35 to 5.72 in males, and 0.47 to 3.21 in females. The weight factors were calculated by dividing the amount of males and females per age group (18–49, 50–64, and 65+) in the study sample with the corresponding age groups of the Dutch general population as provided by the Dutch Central Bureau of Statistics on 1-12-2019 (15).

## Results

### Study Population

The questionnaire was sent to 2,251 panel members, of which 932 (41.1%) filled out the questionnaire. The median age was 67.0 (*IQR* 17) years. A total of 444 (47.6%) males and 488 (52.4%) females took part.

### Frequency and Duration of Experienced Tinnitus

Table 1 shows that 338 of 932 participants (9 missing, 36.3%) experienced tinnitus in the last year. Of those 338, 81 (3 missing, 24.0%) experienced it for <5 min, 64 (3 missing, 18.9%) for 5–60 min, 41 (3 missing, 12.1%) for >60 min or more, and 149 (3 missing, 44.1%) continuously. Answers to questions regarding the duration of the experienced sound were combined with answers to questions regarding frequency. One hundred thirty-two of 216 (61.1%) participants experienced tinnitus continuously, daily or almost daily in the last year. Forty-two of 81 (51.9%) participants experienced tinnitus <5 min every month in the last year (Table 2).

**Table 1:** Duration of tinnitus experience in the last year stratified per mid-decade age groups.

**Age**	**Experience of tinnitus^a^ [*N* (%)]**	**<5 min [*N* (%)]**	**5–60 min [*N* (%)]**	**≥60 min or more [*n* (%)]**	**Continuously [*n* (%)]**
18–24	1 (0.3)	0 (0.0)	1 (1.6)	0 (0.0)	0 (0.0)
25–34	0 (0.0)	0 (0.0)	0 (0.0)	0 (0.0)	0 (0.0)
35–44	13 (3.8)	2 (2.5)	7 (10.9)	2 (4.9)	2 (1.3)
45–54	48 (14.2)	18 (22.2)	7 (10.9)	7 (17.1)	16 (10.7)
55–64	76 (22.5)	20 (24.7)	14 (21.9)	7 (17.1)	35 (23.5)
65–74	130 (38.5)	33 (40.7)	16 (25.0)	17 (41.5)	62 (41.6)
75+	70 (20.7)	8 (9.9)	19 (29.7)	8 (19.5)	34 (22.8)
All	338 (36.3)	81 (24.0)	64 (18.9)	41 (12.1)	149 (44.1)

a *In the last year, nine missing (1.0%) for experience of tinnitus, three missing (0.9%) for duration (<5 min–continuously)*.

**Table 2:** Frequency of tinnitus experience in the last year stratified per duration (Table 1) and mid-decade age groups in amount and percentage *n* (%).

**Age**	**<5 min [** ***n*** **= 81, 0 missing (0%)]**				**5–60 min [** ***n*** **= 61, 3 missing (4.7%)]**				**≥60 min [** ***n*** **= 41, 0 missing (0%)]**				**Continuously [** ***N*** **= 138, 11 missing (7.4%)]**				**Pathologic Tinnitus (*N* = 216)**
	**D**	**W**	**M**	**Y**	**D**	**W**	**M**	**Y**	**D**	**W**	**M**	**Y**	**D**	**W**	**M**	**Y**	
18–24	0 (0.0)	0 (0.0)	0 (0.0)	0 (0.0)	**0 (0.0)**	**0 (0.0)**	0 (0.0)	1 (20.0)	**0 (0.0)**	**0 (0.0)**	**0 (0.0)**	0 (0.0)	**0 (0.0)**	**0 (0.0)**	**0 (0.0)**	0 (0.0)	0 (0.0)
25–34	0 (0.0)	0 (0.0)	0 (0.0)	0 (0.0)	**0 (0.0)**	**0 (0.0)**	0 (0.0)	0 (0.0)	**0 (0.0)**	**0 (0.0)**	**0 (0.0)**	0 (0.0)	**0 (0.0)**	**0 (0.0)**	**0 (0.0)**	0 (0.0)	0 (0.0)
35–44	0 (0.0)	0 (0.0)	2 (4.8)	0 (0.0)	**2 (10.0)**	**3 (15.0)**	1 (6.3)	1 (20.0)	**2 (9.5)**	**0 (0.0)**	**0 (0.0)**	0 (0.0)	**2 (1.5)**	**0 (0.0)**	**0 (0.0)**	0 (0.0)	9 (4.2)
45–54	2 (40.0)	4 (33.3)	11 (26.2)	1 (4.5)	**1 (5.0)**	**3 (15.0)**	3 (18.8)	0 (0.0)	**3 (14.3)**	**2 (20.0)**	**2 (22.2)**	0 (0.0)	**13 (9.8)**	**1 (33.3)**	**0 (0.0)**	1 (50.0)	25 (11.6)
55–64	0 (0.0)	4 (33.3)	9 (21.4)	7 (31.8)	**6 (30.0)**	**6 (30.0)**	0 (0.0)	1 (20.0)	**3 (14.3)**	**2 (20.0)**	**1 (11.1)**	1 (100)	**32 (24.2)**	**2 (66.7)**	**0 (0.0)**	1 (50.0)	52 (24.1)
65–74	0 (0.0)	4 (33.3)	17 (40.5)	12 (54.4)	**4 (20.0)**	**4 (20.0)**	6 (37.5)	2 (40.0)	**8 (38.1)**	**6 (60.0)**	**3 (33.3)**	0 (0.0)	**56 (42.4)**	**0 (0.0)**	**0 (0.0)**	0 (0.0)	81 (37.5)
75+	3 (60.0)	0 (0.0)	3 (7.1)	2 (9.1)	**7 (35.0)**	**4 (20.0)**	6 (37.5)	0 (0.0)	**5 (23.8)**	**0 (0.0)**	**3 (33.3)**	0 (0.0)	**29 (22.0)**	**0 (0.0)**	**1 (100)**	0 (0.0)	49 (22.7)
All	5 (6.2)	12 (14.8)	42 (51.9)	22 (27.2)	**20 (31.3)**	**20 (31.3)**	16 (25.0)	5 (7.8)	**21 (51.2)**	**10 (24.4)**	**9 (22.0)**	1 (2.4)	132 (88.6)	3 (2.0)	1 (0.7)	2 (1.3)	216 (23.2)

*D, Daily; W, Weekly; M, monthly; Y, once or less than once per year. These are written in bold*.

### Numbers of Pathological Tinnitus

We defined 216 (23.2%) of the complete study population (932 participants) as having pathologic tinnitus. When weighted for age and gender, this changed to 195 of 932 participants (21.0%). This resulted in 63.9% of those that experienced tinnitus in the last year (216 of 338). Fifty-two of the 216 pathologic tinnitus participants (24.1%) were between 55 and 64 years of age. The median age of the participants with pathologic tinnitus was 66.5 years (*IQR* 15). One hundred twenty-four (57.4%) of the participants with pathologic tinnitus (*n* = 216) were male.

### Impact of Tinnitus on Daily Life

Tinnitus distress scores measured with the TFI were calculated for the pathologic tinnitus participants. The median TFI score was 16.6 (*IQR* 21.8) (based on 212 participants, 4 missing). Participants who experienced tinnitus daily continuously had the highest median TFI score of 20.4 (*IQR* 29.2) (Table 3). Fifty percent (50.4%) of all pathologic tinnitus participants had a TFI score in the range 0–17 (*n* = 109, 4 missing). One hundred three of 216 pathologic tinnitus participants (47.7%, 4 missing) had a TFI score of 18 or higher. This is 11.1% of the complete sample (*n* = 932). When weighted for age and gender, this changed to 83 of 932 participants (8.9%). On the different TFI subscales, the highest median [43.3 (*IQR* 28.3)] was scored in the subscale: *sense of control* (Table 4).

**Table 3:** Median (*IQR*) TFI scores stratified per mid-decade age group of pathologic tinnitus participants.

**Age**	**TFI median**																	
	**5 to 60 min**				**60 min or more**						**Continuously**						**Pathologic Tinnitus**	
	**D**	**N**	**W**	**N**	**D**	**N**	**W**	**N**	**M**	**N**	**D**	**N**	**W**	**N**	**M**	**N**		**N**
18–24	–	0	–	0	–	0	–	0	–	0	–	0	–	0	–	0	–	0
25–34	–	0	–	0	–	0	–	0	–	0	–	0	–	0	–	0	–	0
35–44	7.4	2^b^	9.2	3^b^	24.4	2^b^	–	0	–	0	25.4	2^b^	–	0	–	0	10.0 (17.8)	9
45–54	1.2	1^a^	8.4	3^b^	26.6	2^*^^b^	19.8	2^b^	5.8	2^b^	15.6 (21.8)	13	12.4	1^a^	–	0	15.0 (21.4)	24
55–64	20.6 (46.6)	6	13.6 (31.4)	6	14.0	3^b^	11.4	2^b^	30.4	1^a^	21.8 (29.3)	32	10.4	2^b^	–	0	19.0 (26.0)	52
65–74	12.2 (11.3)	4	24.6 (38.7)	4	17.4 (19.2)	8	23.4 (21.6)	6	12.8	2^*^^b^	21.2 (35.2)	55^*^	–	0	–	0	20.0 (28.4)	79
75+	11.1 (14.0)	6^*^	10.5 (21.5)	4	19.2 (20.2)	5	–	0	6.7	3^b^	15.6 (31.2)	29	–	0	2.0	1^a^	13.9 (18.8)	48
All	10.0 (12.0)	19^*^	9.8 (24.8)	20	19.2 (18.0)	20^*^	19.4 (13.1)	10	9.4 (20.7)	8^*^	20.4 (29.2)	131^*^	12.4	3^b^	2.0	1^a^	16.6 (21.8)	212

*D, Daily; W, Weekly; M, monthly; Y, once or less than once per year. N = 212 (4 missing)*.

* *One missing*.

a *The real TFI score is presented (not a median)*.

b *IQR could not be calculated*.

**Table 4:** TFI characteristics of pathologic tinnitus participants (*N* = 212, 4 missing).

**TFI characteristic**		***N* (%)**
TFI ranges	0–17	109 (50.4)
	18–31	52 (24.1)
	32–53	26 (12.0)
	54–72	22 (10.2)
	73–100	3 (1.4)
	Missing	4 (1.9)
TFI subscales [median (*IQR*)]	Intrusiveness	26.7 (32.5)
	Sense of control	43.3 (28.3)
	Cognitive	10.0 (30.0)
	Sleep	10.0 (26.7)
	Auditory	20.0 (49.2)
	Relaxation	10.0 (26.7)
	Quality of life	2.5 (20.0)
	Emotional	6.7 (20.0)

On the question “How big a problem is your tinnitus at this moment?” 51 of 216 (23.6%, 1 missing) answered it is not a problem (Table 5). One hundred five of 216 (48.6%, 1 missing) judged their tinnitus to be a small problem, 43 of 216 (19.9%, 1 missing) as a reasonable problem, 12 of 216 (5.6%, 1 missing) as a large problem, and 4 of 216 (1.9%, 1 missing) as a very large problem. One hundred sixty-four of 216 (75.9%, 1 missing) judged their tinnitus to be a small, reasonable, large, or very large problem. This is 17.6% of the total population (164 of 932). When weighted for age and gender, this changed to 147 of 932 participants (15.7%).

**Table 5:** Answers to the question: How big a problem is your tinnitus at this moment? of pathologic tinnitus participants.

**Age**	**No problem**	**Small problem**	**Reasonable problem**	**Large problem**	**Very large problem**
18–24	0 (0.0)	0 (0.0)	0 (0.0)	0 (0.0)	0 (0.0)
25–34	0 (0.0)	0 (0.0)	0 (0.0)	0 (0.0)	0 (0.0)
35–44	2 (3.9)	5 (4.8)	2 (4.7)	0 (0.0)	0 (0.0)
45–54	7 (13.7)	15 (14.3)	3 (7.0)	0 (0.0)	0 (0.0)
55–64	16 (31.4)	22 (21.0)	9 (20.9)	3 (25.0)	2 (50.0)
65–74	16 (31.4)	39 (37.1)	19 (44.2)	4 (33.3)	2 (50.0)
75+	10 (19.6)	24 (22.9)	10 (23.3)	5 (41.7)	0 (0.0)
All	51 (23.6)	105 (48.6)	43 (19.9)	12 (5.6)	4 (1.9)

*One missing (0.5%). Stratified per mid-decade*.

## Discussion

We evaluated the prevalence of tinnitus in the Dutch general population with different cutoffs for its definition. Frequency, duration, and the impact of tinnitus on daily life were individually assessed in an adult sample of inhabitants of the Netherlands.

### Tinnitus Presence

In our study, 36.6% of the participants experienced tinnitus within the last year. Tinnitus was described as *the hearing of e.g., a beep, whistle, sissing, zoom, or another sound without the actual presence of the sound in your surroundings. This can last for a very short amount of time or a whole day*. Only 23.2% (unweighted) [or 21.0% (weighted)] of the participants were defined as having pathologic tinnitus [5–60 min (daily or almost daily or weekly), >60 min or continuously (daily or almost daily or weekly or monthly)]. The difference in these numbers underlines the importance of the exact definition in order to assess prevalence numbers in a population.

This is clearly illustrated in a systematic review by McCormack et al. (1). All included studies were population studies and reported only on adults showing prevalence numbers between 5.1 and 42.7%. Out of 39 included studies, eight different definitions for tinnitus were found. Twenty-six studies used one of the following three definitions: “*tinnitus lasting for more than 5 min at a time” (12 studies, prevalence ranged between 11.9 and 30.3%), “do you have tinnitus” (5 studies, prevalence ranged between 10.1 and 22%)*, or “*within the last year did you experience tinnitus” (9 studies, prevalence ranged range* 6.1 and 24.6%). Even with the most commonly used definition, *tinnitus lasting for more than 5 min at a time*, the reported prevalence numbers ranged between 11.9 and 30.3% in included studies (1). Our prevalence number of 23.3% (unweighted) or 21.0% (weighted) of cases with pathologic tinnitus falls within that range. McCormack et al. reported that in those studies similar study groups in terms of age and a similar definition were used (>*5 min)*, in which the prevalence numbers still varied largely (e.g., for people aged 60–70 between 13.3 and 35.5%) (1). This, again, stresses the importance of a uniform way to study tinnitus, with a similar question and similar response options.

### The Impact of Tinnitus on Daily Life

For the present study, we based our definition of the presence of pathologic tinnitus on the combination of duration and frequency. However, the mere presence of tinnitus does not explain the impact of tinnitus on a person's daily life. In our study, we used a multi-item questionnaire, the TFI, to measure the impact of tinnitus on daily life. A score between 0 and 17 can be interpreted as “not a problem” (14). In our study, a majority of the participants (50.4%), defined with pathologic tinnitus, had a score between 0 and 17 on the TFI. With a TFI score of 18 or more, the tinnitus can be interpreted as at least a small problem. We found that 49.7% of the 216 pathologic tinnitus participants [or 11% (unweighted) (103 out of 932) out of the total participants or 8.9% (weighted) (83 of 932)] had a TFI score of 18 or more. This is similar to the study by Oosterloo et al., in which they studied a Dutch population sample of older adults (≥50 years) out of 2020. They found that for 12.3% of the people with tinnitus, a positive score was noted on the single question *does the tinnitus interfere with daily life?* (4). This underlines once again that even our definition of pathologic tinnitus entailing duration and frequency does not seem to correlate with the impact of tinnitus on daily life (as measured by the TFI). This might suggest that in order to identify people with pathologic tinnitus, one should rely on validated tinnitus measures of impact on daily life after people are indicated as having tinnitus based on the experienced sounds (1).

However, the use of validated measures in population studies is difficult because of logistical issues due to the lengthiness of the questionnaires. Contrarily, Biswas et al. propose to use a single-item question to assess tinnitus severity: “over the past year, how much do these noises in your head or ears worry, annoy or upset you when they are at their worst?” (16). Interestingly, we also asked the participants a single-item question to assess severity: “how big a problem is your tinnitus at this moment?” If combined, we found that 164 of 216 (75.9%) judged tinnitus to be at least a small problem. This was 17.6% (unweighted) or 15.7% (weighted) of all 932 participants. The difference in prevalence numbers between measuring the impact of tinnitus with a multi-item questionnaire or a single-item question again shows the importance of reaching consensus on how to handle this issue. Perhaps we have still yet to find the optimal tool to measure the impact of tinnitus on daily life for similar study settings. Still, we only asked participants to fill out both the TFI and the question “how big a problem is your tinnitus?” if they met our definition of having pathologic tinnitus. It would also be interesting to see if those who did not meet our definition of pathologic tinnitus, but did experience tinnitus, considered their tinnitus to be a problem.

## Strengths and Limitations

Several strengths and limitations are applicable to this study. The first strength is that the study was performed in a sample of the Dutch population, rather than a selected cohort. The second includes the extensiveness and specificity of questioning regarding the tinnitus prevalence. Multiple factors were included in the definition and related to prevalence. The gender distribution of the participants to the questionnaire was similar to that of the Dutch population in 2020 (15). However, a limitation of our study is that the age distribution of the respondents was not representative of the Dutch population (15). The higher age in our sample and the knowledge that tinnitus prevalence increases with age could mean that our numbers overestimated the prevalence numbers in the real population. This is also illustrated by the lower prevalence numbers when weighted for age and gender, which probably better reflect the prevalence in the population. Although we had 932 participants, a response rate of 41.4% was reached, which could result in selection bias. Nivel consumer panel members sign up to receive questionnaires on all sorts of healthcare topics, which resulted in response rates of 50–60% historically. The low response rate to the current questionnaire could be related to the lengthiness of the complete questionnaire (eight pages), or the topic of the questionnaire.

## Recommendations

The lack of a clear definition and subsequently the lack of prevalence numbers in general populations around the world are two important obstacles that hinder the search for a curative treatment (6, 16). The difficulties of defining tinnitus with and without suffering have recently been addressed by the Tinnitus Research Initiative (TRI) in a conceptual book chapter (17, 18). The authors propose a different definition for tinnitus and tinnitus disorder. They argue that tinnitus becomes a tinnitus disorder “when associated with emotional distress, cognitive dysfunction, and/or automatic arousal, leading to behavioral changes and functional disability” [(13), p. 8]. Next, they also advise frequency and duration to be used in the definition of tinnitus; they advise that tinnitus should occur for a minimum of 5 min a day on the majority of days. In order to find a treatment for tinnitus, we believe that tinnitus research needs to go back to its basics. A clear standardized definition of pathologic tinnitus is the obvious starting point. Only then can true comparisons between different study populations be made (1). We therefore encourage all researchers to adapt the definitions as recently proposed by the TRI (17).

## Conclusion

In this study, we found that 36.6% of all participants experienced tinnitus for whatever amount of time over the last year. Of those 23.2% that met our definition of pathologic tinnitus, which was based on a combination of duration and frequency over the last year. When weighted for age and gender, this decreased to 21.0%. A percentage of 48.6% of the pathologic tinnitus participants had a TFI score which indicates that they consider their tinnitus to be at least a small problem [11% of the total sample (unweighted) or 8.9% (weighted)]. This study demonstrates that tinnitus prevalence numbers vary with different definitions. It therefore highlights the need to use a uniform definition of tinnitus to compare outcomes.

## Data Availability Statement

The datasets generated for this article are not readily available because the Dutch Health Care Consumer Panel has a program committee, which supervises processing the data of the Dutch Health Care Consumer Panel and decides about the use of the data. This program committee consists of representatives of the Dutch Ministry of Health, Welfare and Sport, the Health Care Inspectorate, Zorgverzekeraars Nederland (Association of Health Care Insurers in the Netherlands), the National Health Care Institute, the Federation of Patients and Consumer Organisations in the Netherlands, the Dutch Healthcare Authority, and the Dutch Consumers Association. All research conducted within the Consumer Panel has to be approved by this program committee. The committee assesses whether a specific research fits within the aim of the Consumer Panel, which strengthens the position of the healthcare user. Requests to access the datasets should be directed to the corresponding author.

## Ethics Statement

Ethical review and approval was not required for the study on human participants in accordance with the local legislation and institutional requirements. Written informed consent for participation was not required for this study in accordance with the national legislation and the institutional requirements.

## Author Contributions

MR, AS, RS, and IS contributed to the conception. MR, AB, RS, AS, and IS contributed to the design of the study. AB and JJ contributed to the data collection. MR, AS, and IS contributed to the methodology and statistical analyses. MR wrote the first version of the manuscript. All authors contributed to the manuscript revision, read, and approved the submitted version.

## Conflict of Interest

The authors declare that the research was conducted in the absence of any commercial or financial relationships that could be construed as a potential conflict of interest.

## Publisher's Note

All claims expressed in this article are solely those of the authors and do not necessarily represent those of their affiliated organizations, or those of the publisher, the editors and the reviewers. Any product that may be evaluated in this article, or claim that may be made by its manufacturer, is not guaranteed or endorsed by the publisher.
